# Contribution of *WUSCHEL-*related homeobox (*WOX*) genes to identify the phylogenetic relationships among *Petunia* species

**DOI:** 10.1590/1678-4685-GMB-2016-0073

**Published:** 2016-10-20

**Authors:** Ana Lúcia Anversa Segatto, Claudia Elizabeth Thompson, Loreta Brandão Freitas

**Affiliations:** 1 Laboratory of Molecular Evolution, Department of Genetics, Universidade Federal do Rio Grande do Sul (UFRGS), Porto Alegre, RS, Brazil; 2 Center for Biotechnology, Department of Molecular Biology and Biotechnology, Universidade Federal do Rio Grande do Sul (UFRGS), Porto Alegre, RS, Brazil

**Keywords:** molecular phylogeny, recently diverged species, developmental genes

## Abstract

Developmental genes are believed to contribute to major changes during plant evolution, from infrageneric to higher levels. Due to their putative high sequence conservation, developmental genes are rarely used as molecular markers, and few studies including these sequences at low taxonomic levels exist. *WUSCHEL*-related homeobox genes (*WOX*) are transcription factors exclusively present in plants and are involved in developmental processes. In this study, we characterized the infrageneric genetic variation of *Petunia WOX* genes. We obtained phylogenetic relationships consistent with other phylogenies based on nuclear markers, but with higher statistical support, resolution in terminals, and compatibility with flower morphological changes.

## Introduction

Flowering plants exhibit flower shapes that can be very different from one species to another in terms of their architecture. Adaptive radiation, referred as the rapid species diversification with increased phenotypic diversity (Schluter, 2000), has been proposed as an explanation for the high diversity observed in several plant species in some regions, especially those areas that have experienced rapid climatic or geological changes (Hughes and Eastwood, 2006). Species complexes originated from unstable areas could be of particular interest for evolutionary studies, but could also constitute a challenge. The morphological and phylogenetic species circumscription could be hampered by evolutionary processes such as, for example, recent or ancient gene flow (Hey, 2010), incomplete lineage sorting, or horizontal gene transfer (Knowles and Carstens, 2007). *Petunia* Juss. has undergone rapid diversification during the Pleistocene climatic changes (Lorenz-Lemke *et al.*, 2010), and molecular phylogenies did not resolve all morphological species (Kulcheski *et al.*, 2006; Chen *et al.*, 2007; Reck-Kortmann *et al.*, 2014).

Morphologically, the *Petunia* species may be divided into two main groups according to the corolla tube length. These groups have been supported in a multilocus phylogeny (Reck-Kortmann *et al.*, 2014), despite the limited sequence divergence among species and indeterminate terminal positions. The long corolla tube group includes species with different floral syndromes (sphingophily, melittophily, and ornithophily) and several divergent morphological traits related to the pollinators, whereas among the species with a short corolla tube all are bee-pollinated (melittophily) and present fewer morphological differences among their flowers. Despite the morphologically homogeneous flowers, short corolla tube species present differences that could be attributed to developmental genes, such as corolla shape and adnation of floral pieces. All these attributes elect *Petunia* as one of the most diverse flower genera in Solanaceae Juss. (Knapp, 2010).

The *WUSCHEL*-related homeobox (*WOX*) gene family was first identified in *Arabidopsis thaliana* (L.) Heynh and posteriorly characterized in several plant species (Constanzo *et al.*, 2014). Despite the *WOX* gene family being involved in flower development, these genes are present in algae but not in other eukaryotes outside the plant kingdom (Deveaux *et al.*, 2008; Graaff *et al.*, 2009). Studies have shown that the *WOX* gene family in the *Petunia* is dynamically involved in flower morphology and inflorescence determination, primarily coordinating cell proliferation (Stuurman *et al.*, 2002; Rebocho *et al.*, 2008; Vandenbussche *et al.*, 2009; Constanzo *et al.*, 2014). The diversity of flower morphology in *Petunia,* together with the availability of published studies describing *WOX* genes in this genus, led us to choose this species to characterize *WOX* variability at an infrageneric level. Seven *WOX* genes were described in the genome of *P.* x *hybrida* (Hook.) Vilm. (Constanzo *et al.*, 2014), and these genes are involved in different steps of flower and inflorescence architecture and development. For example, the *MAW* gene (*MAEWEST*), an ortholog of *AtWOX1* that is required for petal and carpel fusion and lateral growth of the leaf blade (Vandenbussche *et al.*, 2009), presents different expression levels through the different developmental stages in wild *Petunia* species with short (*P. inflata* R.E.Fr.) and long corolla tubes [*P. axillaris* (Lam.) Britton, Sterns & Poggenb.], indicating the possible participation of *MAW* in the regulatory network that leads to different corolla morphologies (Segatto *et al.*, 2013). Other well-characterized *Petunia WOX* genes are *PhEVG* (*EVERGREEN*) and *PhSOE* (*SISTER OF EVERGREEN*), both closely related to the *AtWOX8* and *AtWOX9* of *A. thaliana. PhEVG* and *PhSOE* were originated from a relatively recent event of duplication in *P.* x *hybrida*, but while *PhEVG* is exclusively expressed in incipient lateral inflorescence meristems and it is essential for the specification of cymose inflorescence type in *Petunia* (Constanzo *et al.*, 2014), *PhSOE* has different patterns of expression and it is most likely involved in the development of the shoot apical meristem (Rebocho *et al.*, 2008). In this study, the *WOX* genes were used to better understand the *Petunia* species evolutionary relationships.

## Material and Methods

**Figure 1:**
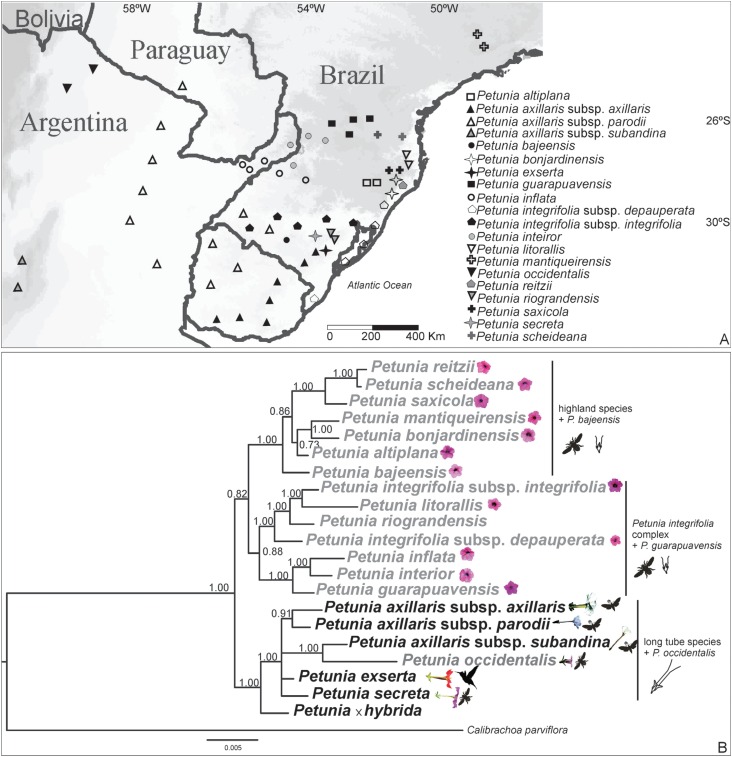
Bayesian inference phylogeny and geographic distribution of *Petunia* species. (A) Schematic representation of *Petunia* geographic distribution. (B) Bayesian phylogenetic tree considering the intron/exon *WUSCHEL*-related homeobox gene sequences in *Petunia* species. The posterior probabilities are indicated above the branches. The short corolla tube species names are in gray, and the long corolla tube species are in black. Species pollinators are represented (bee, hawk-moth, and hummingbird).

We analyzed partial sequences from 21 *Petunia* taxa (according to Ando *et al.*, 2005a) and *Calibrachoa parviflora* (Jussieu) D’Arcy collected in southern Brazil, Argentina, and Uruguay (Figure 1A and Table S1) for the *WOX* genes of *P.* x *hybrida* (*PhWUS, PhWOX1/MAW, PhWOX2, PhWOX3/PRS, PhWOX4, PhEVG, and PhSOE*). The primers, designed with Primer3 0.4 (Rozen and Skaletsky, 2000) (Table S2), were 20–24 base pairs (bp) long and shared 100% homology with the *P.* x *hybrida* sequences. The TM of each primer was ca. 60°C. Polymerase chain reaction (PCR) amplifications were performed in 25 μL reactions consisting of 1 unit of Platinum *Taq* polymerase (Invitrogen, Carlsbad, CA, USA), 1X Platinum *Taq* polymerase buffer (Invitrogen), 0.2 mM of each dNTP, 0.2 mM MgCl_2_, 0.2 μM of each primer, 5% of dimethyl sulfoxide (DMSO), and 20–50 ng of genomic DNA as a template. The following PCR conditions were used: 94 °C for 3 min for the initial denaturation of the fragments, 35 cycles of 30 s at 94 °C, 55 °C, and 72 °C each, with a final extension step of 10 min at 72 °C to complete the synthesis. The PCR products were purified using 20% polyethylene glycol (Dunn and Blattner, 1987) and sequenced in a MegaBACE 1000 DNA Analysis System (GE Healthcare, Biosciences, Pittsburgh, PA, USA) using the ET Terminator Kit (GE Healthcare) according to the manufacturer’s instructions. As different members of the *WOX* gene family are divergent outside the homeodomain region (Deveaux *et al.*, 2008), this enabled us to sequence the genes in *Petunia* without cloning, by positioning the primers in adjacent regions of the homeodomain.

The obtained sequences were aligned using MUSCLE (Edgar, 2004) software as implemented in MEGA6 (Tamura *et al.*, 2013) and then manually edited. We compared exon (including the homeodomain) and intron sequence diversity and their resulting phylogenies to characterize the *WOX* gene variation inside *Petunia* (see Table S1 for GenBank accession and voucher numbers). Heterozygous sites were standardized by the more frequent nucleotides in the position in all individuals and in all analyses. A site was identified as heterozygous when double peaks occurred in the identical position in both strands, with the weakest signal reaching at least 25% of the strength of the strongest (Fuertes Aguilar and Nieto Feliner, 2003). The nucleotide heterozygous sites never induced amino acid substitutions. *Calibrachoa parviflora* sequences were used as outgroups. We used jMODELTEST 2.1.4 (Posada, 2008) to determine the best evolutionary models for the DNA alignment, based on the Akaike Information Criterion (AIC). The best-fit model was the GTR (Generalized Time Reversible) with a discrete gamma-distribution of rate variation across nucleotide sites. Bayesian Inference (BI) analyses were conducted using the MRBAYES 3.2.2 program (Ronquist and Huelsenbeck, 2003) with the previously described DNA evolutionary models and four chains were run for 10,000,000 generations, with 25 % genealogies discarded as burn-in. The intron, exon, and total (intron + exon) *WOX* trees were estimated. The C + G content, number of variable sites, and average nucleotide diversity per site (π) were calculated using DNASP 5.00.03 (Rozas *et al.*, 2003).

## Results

The first introns of the genes *WUS, WOX1,* and *WOX4* of the *Petunia* species were sequenced and concatenated, leading to the alignment of 792 bp. The exon-concatenated alignment was 2,454 bp long and consisted of partial sequences of the first exon of *WUS, WOX2,* and *WOX3*, partial sequences of the first and second exon of *WOX1* and *WOX4*, and partial sequence of the second exon of *SOE* and *EVG* genes. The sequenced exon regions contained the homeodomain region for all previously described genes. Regarding the homeodomain region, almost all *WOX* genes in *Petunia* shared the conserved sequence NVFYWFQN in the homeodomain helix region and only *SOE* had the sequences NVFLLVSN or KCFLLVSN (Figure S1) that increase the percentage of neutral and aliphatic amino acids. The concatenated exons of the *WUSCHEL-*related homeobox exhibited higher G + C content than the concatenated introns and more polymorphic sites, but lower nucleotide diversity on average per site (Table 1). The intron, exon, and intron + exon datasets produced BI phylogenies with very similar topology (Figure S2), and the intron + exon tree presented the highest posterior probabilities for all branches (Figure 1B) that were similar to other phylogenetic propositions. Two main clades that correspond to species with short and long corolla tubes were recovered in both intron + exon and intron-only trees, whereas in the exon-based tree the species presenting long corolla tube did not group. The majority of relationships between sister species was maintained in all trees, with exceptions corresponding to low-supported branches. *Petunia* x *hybrida* was positioned as a sister group of short tube species in the exon-only and intron-only trees and as a sister group of the long tube species in the intron + exon tree. In the intron + exon tree, *Petunia bajeensis* T.Ando & Hashim., a microendemic species that occurs at altitudes below 200 m, was the sister species of the group living in altitudes higher than 900 m. *Petunia guarapuavensis* T.Ando & Hashim. was closely related to *P. inflata* R.E.Fr. and *P. interior* T.Ando & Hashim. in trees obtained with all datasets, even though *P. guarapuavensis* is morphologically more similar to *P. scheideana* L.B.Sm. & Downs and does not occur in the same geographic region than *P. interior* and *P. inflata* (Figure 1A). This is the first time in *Petunia* phylogeny studies that the species *P. riograndensis* T.Ando & Hashim. and *P. integrifolia* subsp. *integrifolia* and the species *P. littoralis* L.B.Sm. & Downs and *P. integrifolia* subsp. *depauperata* (R.E.Fr.) Stehmann did not form homogeneous groups, respectively. Contrarily to the expected, the infraspecific taxa did not form closely related groups and this was observed in *P. integrifolia* and in *P. axillaris* subspecies, respectively. *Petunia occidentalis* R.E.Fr., a short corolla tube flowering species, was grouped into the long corolla tube clade (Figure 1B).

**Table 1:** *Petunia WOX* genes sequence polymorphisms.

	Exons	Introns	Exons + introns
G + C content	39 %	26 %	37 %
Number of variable sites	108	34	142
Average number of nucleotide diversity per site (π)	0.012 ± 0.003	0.026 ± 0.005	0.014 ± 0.003

## Discussion

In this work, we present a detailed evolutionary characterization of *WOX* genes in wild *Petunia* species, aiming to highlight the contribution of the *WUSCHEL* gene family to phylogenetic studies. As a result of their high phenotypic diversity and rapid speciation (Lorenz-Lemke *et al.*, 2010), *Petunia* species are an ideal model to examine evolutionary innovations and adaptation.

In *Petunia*, two main clades associated with corolla tube length are consistently obtained in molecular marker-based phylogenies (Chen *et al.*, 2007; Kriedt *et al.*, 2014; Reck-Kortmann *et al.*, 2014), despite the low support in internal branches and uncertainty of some terminal positions. Flower morphology directly correlates with pollinator shift and has impacted *Petunia* diversification (Fregonezi *et al.*, 2013). Similar results are consistently shown for several plant groups in which changes in the pollination system are involved in increasing the diversification rates at the macroevolutionary level (Kay and Sargent, 2009) and enlarging the polymorphisms at the microevolutionary scale (Bradshaw and Schemske, 2003; Dyer *et al.*, 2007; Sheehan *et al.*, 2016). Pollinator shift could be caused by few phenotypic modifications making it a rapid mechanism of reproductive isolation, but it is rare and probably occurs in highly specialized taxa. The adaptation to different environmental conditions or habitats, coupled with reproductive isolation by floral isolation generates different selection pressures and can determine the success of the new lineage (Chase *et al.*, 2010). The most contrasting phenotypic characteristics in *Petunia* are between the two main clades and within the long corolla tube clade. While in the short corolla tube clade species share the same pollinator, in the long corolla tube clade there are three different floral syndromes. It is probable that pollination change influenced *Petunia* diversification in different ways among and within clades. The differential expression of *WOX* gene is associated with flower development and differentiation in at least two *Petunia* species with contrasting corolla tube length (Segatto *et al.*, 2013).

Here, all datasets used to build the phylogenetic trees showed significant improvement at the branches support in comparison with previous used markers and analyses, indicating the utility of the *WOX* genes as markers for understanding species evolution in *Petunia*. Considering the complete dataset (intron + exon), we observed two highly supported clades corresponding to short and long corolla tube length.

At a second level, two well-supported clades appear, dividing the short and purple corolla tube and bee-pollinated species: the first corresponds to highland species, plus *P. bajeensis,* and the second is composed of the *P. integrifolia* group (all taxa with morphological traits similar to *P. integrifolia* and described, at least once, as infraspecific taxon) plus *P. guarapuavensis* (Figure 1B). *Petunia bajeensis*, a species that occurs just in lowlands, was the sister group of the highland species (Figure 1B), confirming previous findings of phylogenies based on combined nuclear and plastid markers (Reck-Kortmann *et al.*, 2014). However, such relationships were not observed in phylogenies obtained through plastid sequence analysis (Lorenz-Lemke *et al.*, 2010) or plastid RFLP (Ando *et al.*, 2005a), in which *P. bajeensis* is placed in the lowland group of species, or those based only on nuclear sequences (Chen *et al.*, 2007; Kriedt *et al.*, 2014), in which *P. bajeensis* is placed in the highland group, with different sister species. All highland species share morphological traits with *P. bajeensis*, especially the stamen filaments adnated up to half of the corolla tube (Stehmann *et al.*, 2009). Events of hybridization and/or incomplete lineage sorting, added to different coalescence times of the markers as discussed below, could explain the contrasting phylogenetic results. However, *P. bajeensis* occurs in lowlands and in the middle of *P. integrifolia* subsp. *integrifolia* distribution, and it is difficult to draw a biogeographic scenario in which this species would have the last common ancestor with a highland species, based on the data we have at the moment.

The sub-clade corresponding to the *P. integrifolia* group plus *P. guarapuavensis* was observed for the first time, and the presence of *P. guarapuavensis* in this group finds support in the extensive morphological similarity between that species and *P. inflata* (Ando and Hashimoto, 1995). Despite that *P. guarapuavensis* and *P. scheideana* share several morphological traits and have a similar geographic distribution (Figure 1A), they occupy different branches in molecular biology-based analyses (Chen *et al.*, 2007; Lorenz-Lemke *et al.*, 2010; Reck-Kortmann *et al.*, 2014). Ando *et al.* (2005b) suggested as valid the taxa *P. integrifolia* subsp. *depauperata, P. littoralis, P. riograndensis,* and *P. integrifolia* subsp. *integrifolia* because of the differences in their floral traits, but Stehmann and Bohs (2007) proposed grouping all individuals with morphology corresponding to *P. integrifolia* subsp. *depauperata* and *P. littoralis* under *P. integrifolia* subsp. *depauperata.* Posteriorly, Stehmann *et al.* (2009) suggested also the union of *P. riograndensis* and *P. integrifolia* subsp. *integrifolia* under the latter taxa, justifying this choice with the association of several characteristics to the different environments where these plants grow. Previous evolutionary approaches (Longo *et al.*, 2014; Reck-Kortmann *et al.*, 2014; Ramos-Fregonezi *et al.*, 2015) have supported the suggestions of Stehmann and Bohs (2007) and Stehmann *et al.* (2009), but the genetic variation found in *WOX* genes reinforces the proposition of Ando *et al.* (2005a,b), this indicating the need for more studies including these different morphologies.

In the second main clade, *Petunia occidentalis,* which has a short corolla tube, was grouped with the long corolla tube species. Previous studies observed similar phylogenies using nuclear sequences only (*FLAVONOID-3’,5’-HYDROXYLASE* gene, Chen *et al.*, 2007; *Tnt1* mobile elements, Kriedt *et al.*, 2014) and nuclear + plastid markers (Reck-Kortmann *et al.*, 2014). *Petunia occidentalis* presents a disjunct geographical distribution compared to other short corolla tube *Petunia* species (Figure 1). Therefore, it is not surprising that *P. occidentalis WOX* genes exhibit a distinct evolutionary history. All *Petunia* phylogenies based on nuclear markers show a more recent common ancestor between *P. occidentalis* and the long corolla tube group. Different gene trees can diverge as a consequence of several factors, including horizontal transfer, lineage sorting, gene duplication, and extinction events (Maddison, 1997; Nichols, 2001).

Molecular phylogenies based on plastid-derived markers (Ando *et al.*, 2005a; Kulcheski *et al.*, 2006; Lorenz-Lemke *et al.*, 2010) have proposed that the separation among *Petunia* species was related to the altitude of their occurrence, independent of corolla morphology. Nuclear and plastid genes have different coalescence times and, generally, organelle markers are geographically structured and more easily shared by different species (Chan and Levin, 2005; Mir *et al.*, 2009), as observed in some *Petunia* species (Segatto *et al.*, 2014). Therefore, it is not surprising that the trees based on *WOX* genes indicate an evolutionary history that is compatible with others obtained from nuclear information. The specific clades presented are contingent on corolla tube length, particularly because *WOX* genes are involved in floral development and, at least with respect to the *MAW* gene, are differentially expressed in *Petunia* species of different floral morphology (Segatto *et al.*, 2013).

All *WOX* genes, including *SOE,* were most likely present in the last common ancestor of the genus. The use of *WOX* genes as molecular markers to reconstruct *Petunia* phylogeny resulted in a better-supported tree that was congruent with other published phylogenies for nuclear genes. The structure of *WOX* proteins has also contributed to the understanding of molecular evolution and function of these genes in *Petunia* and in the whole Solanaceae family, as well (Stuurman *et al.*, 2002; Rebocho *et al.*, 2008; Vandenbussche *et al.*, 2009; Tadeg *et al.*, 2011; Segatto *et al.*, 2013). In this context, we found a modification in the amino acid content of helix three of the homeodomain region in *Petunia SOE* genes. This helix contacts DNA and has a high level of basic amino acids, which makes it potentially important for nuclear localization (Li *et al.*, 2014). *SOE* is not functionally characterized in *Petunia* but is expressed on the vegetative apex between the shoot apical meristem and leaf primordia, and later in the outermost cells of the placenta where the ovules are formed (Rebocho *et al.*, 2008). SOE is also expressed in the basal end of young embryos, similar to *AthWOX9* and *AthWOX8* (Haecker *et al.*, 2004; Rebocho *et al.*, 2008). The amino acid substitution in helix three of the homeodomain may influence the *SOE* interaction with DNA and/or other transcription factors. Until now, no other *WOX* genes have been described with this modification in helix three. Moreover, this region is strongly conserved in all other *WOX* genes. Consequently, the *SOE* gene warrants further study to evaluate the effects of changes in helix three, cellular localization, and interaction with DNA and other transcription factors, which are, according to evolutionary developmental biology, master keys in evolution. Thus, the characterization of these transcription factors at different taxonomic levels is important to determine sequence conservation, reveal response to different selective pressures, and gene losses after species divergence, resulting in differences in morphological phenotype.

In this study, we characterized the *WOX* transcription gene family in the genus *Petunia* and demonstrated the potential for the use of these genes as molecular markers to reconstruct infrageneric phylogenies. The use of species with significant morphological diversity in the study of the evolutionary dynamics of developmental genes is an interesting research strategy that can be expanded to other developmental genes and botanical families.

## Supplementary material

The following online material is available for this article:

Figure S1 - Alignment of the *Petunia* homeodomain region of *WUSCHEL*-related homeobox genes.

Figure S2 - Bayesian inference phylogenies of *WUSCHEL*-related homeobox gene sequences in *Petunia* species.

Table S1 - *Petunia WUSCHEL*-related homeobox gene sequence information.

Table S2 - Sequences of the primers used in this work.
