# Supplementary material for: Contribution of WUSCHEL-related homeobox (WOX) genes to identify the phylogenetic relationships among Petunia species
Source: Genet Mol Biol. 2016 Oct 20;39(4):658–64. doi: 10.1590/1678-4685-GMB-2016-0073 (PMC5127159; doi:10.1590/1678-4685-GMB-2016-0073)
Supplement: Supplementary file 1 [file 1415-4757-gmb-1678-4685-GMB-2016-0073-Suppl03.pdf]

Table S1 - *Petunia* species for which the *WOX* genes were sequenced and used in the phylogenetics analysis, GenBank numbers and vouchers or sampling geographic information

Species (abbreviated name)	GenBank accession numbers for each gene							Voucher or sampling information
	WUS	*WOX1*	*WOX2*	*WOX3*	*WOX4*	*EVG*	*SOE*	
P. altiplana T. Ando and Hashim. (ALT)	KF928399	KF928347	KF951580	KJ152506	KF928372	KJ159135	KJ186983	BHBC[a] 87269<br>BHCB 104859
P. axillaris (Lam.) Britton, Sterns and Poggenb. ssp. *axillaris* (AXI)	KF928403	KF928351	KF951584	KJ152510	KF928376	KJ159139	KJ186987	ICN 164594[b]
P. axillaris ssp. *parodii* (Steere) Cabrera. (PARO)	KF928404	KF928352	KF951585	KJ152511	KF928377	KJ159140	KJ186988	ICN 158367
P. axillaris ssp. *subandina* T. Ando. (SUB)	KF928412	KF928360	KF951583	KJ152509	KF928385	KJ159138	KJ186986	BHCB 140429
P. bajeensis T. Ando and Hashim. (BAJ)	KF928406	KF928354	KF951587	KJ152513	KF928379	KJ159142	KJ186990	BHCB 002711<br>BHCB 102128
P. bonjardinensis T. Ando and	KF928416	KF928364	KF951593	KJ152520	KF928389	KJ159148	KJ186997	28° 29' 00.39'' S

Hashim. (BONJ)								49° 53' 32.09"W
P. exserta Stehmann. (EXSE)	KF928401	KF928349	KF951582	KJ152508	KF928374	KJ159137	KJ186985	ICN 158542
P. guarapuavensis T. Ando and Hashim. (GUA)	KF928393	KF928341	KF951574	KJ152500	KF928366	KJ159129	KJ186994	BHCB 96617
P. inflata R. E. Fr. (INF)	KF928394	KF928342	KF951575	KJ152501	KF928367	KJ159130	KJ186978	BHCB 127297
P. integrifolia (Hook.) Schinz and Thell. ssp. *integrifolia* (INTE)	KF928415	KF928363	KF951592	KJ152519	KF928388	KJ159147	KJ186996	BHCB 79876<br>BHCB 85218
P. integrifolia ssp. *depauperata* R. E. Fr. (DEPA)	KF928396	KF928344	KF951577	KJ152503	KF928369	KJ159132	KJ186995	30° 20’ 26.63” S<br>50° 16’ 4.48”W
P. interior T. Ando and Hashim. (TERI)	KF928417	KF928363	KF951594	KJ152521	KF928388	KJ159149	KJ186998	BHCB 114598
P. littoralis L. B. Sm. and Downs. (LITO)	KF928414	KF928362	-	KJ152518	KF928387	KJ159131	KJ186979	HUEFS[c] 75262[c]
P. mantiqueirensis T. Ando and Hashim. (MANT)	KF928397	KF928345	KF951578	KJ152504	KF928370	KJ159133	KJ186981	BHCB 78269<br>ESA[d] 101033
P. occidentalis R. E. Fr. (OCI)	KF928405	KF928353	KF951586	KJ152512	KF928378	KJ159141	KJ186989	Urundel, Salta, Argentina, 1988

P. reitzii L. B. Sm. and Downs. (REIT)	KF928408	KF928356	KF951589	KJ152515	KF928381	KJ159144	KJ186992	BHCB 002716<br>BHCB 80073
P. riograndensis T. Ando and Hashim. (RIO)	KF928395	KF928343	KF951576	KJ152502	KF928368	KJ159131	KJ186979	BHCB 75078
P. saxicola L. B. Sm. and Downs. (SAXI)	KF928407	KF928355	KF951588	KJ152514	KF928380	KJ159143	KJ186991	BHCB 002717<br>BHCB 96668
P. scheideana L. B. Sm. and Downs. (SCHE)	KF928410	KF928358	KF951591	KJ152500	KF928383	KJ159129	KJ186994	HUEFS 0075241<br>HUEFS 75241
P. secreta Stehmann & Semir. (SEC)	KF928400	KF928348	KF951581	KJ152507	KF928373	KJ159136	KJ186984	30° 32' 45.92" S<br>53° 33' 00.98"W
P. hybrida (Hook.) Vilm. (VARI)	KF928398	KF928346	KF951579	KJ152505	KF928371	KJ159134	KJ186982	BHCB 143256
Calibrachoa parviflora (Juss.) D'Arcy (PARV)	KF928409	KF928357	KF951590	KJ152516	KF928382	-	KJ186993	CESJ[e] 45735

[a]BHCB- Herbarium of Departamento de Botânica, Universidade Federal de Minas Gerais.; [b]ICN- Herbarium of Instituto de Biociências, Universidade Federal do Rio Grande do Sul; [c]HUEFS- Herbarium of Universidade Estadual de Feira de Santana ; [d]Herbarium of Escola Superior de Agricultura Luiz de Queiroz; [e]Herbarium Leopoldo Krieger
