# Supplementary material for: Contribution of WUSCHEL-related homeobox (WOX) genes to identify the phylogenetic relationships among Petunia species
Source: Genet Mol Biol. 2016 Oct 20;39(4):658–64. doi: 10.1590/1678-4685-GMB-2016-0073 (PMC5127159; doi:10.1590/1678-4685-GMB-2016-0073)
Supplement: Supplementary file 2 [file 1415-4757-gmb-1678-4685-GMB-2016-0073-Suppl04.pdf]

Table S2 - Primers used to amplify *WOX*-described genes in *Petunia ×hybrida* in wild *Petunia* species

Gene	Primer foward 5'-3'	Primer reverse 5'-3'
WUS	WUSE1F (AGATGGTAGCAACAAAAACAACAG)	WUSE2R (ACTGCTAGGACACCATGAGAAGA)
WOX1	PMAWhibrF (ATGTGGATGATGGGTTACAATGAC)	WOX1E2-R1 (AATTGTTACGGGACTGCTCATC)
WOX2	WOX2E1-F (GGATATGTTATCTTCTGGTGGAAC)	WOX2E1-R (CACGATTGAAATAAGCCAAACTC)
WOX3	WOX3E1-F (TTACAAGACCAACAAGATGGAGTC)	WOX3E1-R (GGTGTTGAGCTAAAATTTGCTGTT)
WOX4	WOX4E1-F (CTACTCCCTCACTCTCACTTGGTT)	WOX4E2-R (GACTATGGCTGGTGGTGTTCTTG)
EVG	EVGE1-F (CACTTCAGTGCCAGCAATATG)	EVGE2-R (AGGAGCTGAAGGAACAATGTAATC)
SOE	SOEE1-F (CCTTGCGATTCTCACCATCATACA)	SOEE2-R (TCACATGATGAGAAGAAGAAAAGC)
