# Supplementary material for: Contribution of WUSCHEL-related homeobox (WOX) genes to identify the phylogenetic relationships among Petunia species
Source: Genet Mol Biol. 2016 Oct 20;39(4):658–64. doi: 10.1590/1678-4685-GMB-2016-0073 (PMC5127159; doi:10.1590/1678-4685-GMB-2016-0073)
Supplement: Supplementary file 3 [file 1415-4757-gmb-1678-4685-GMB-2016-0073-Suppl01.pdf]

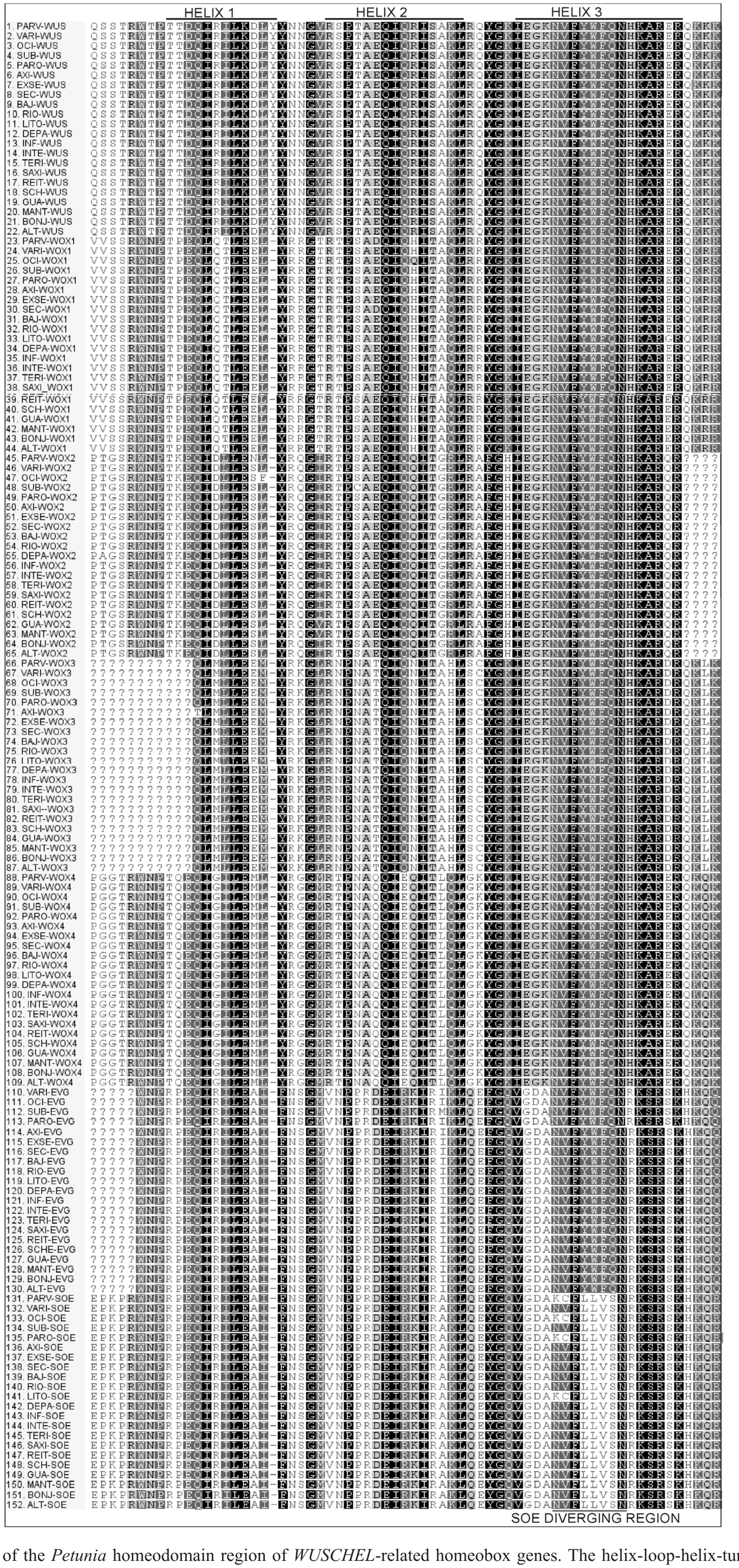

Figure S1 - Alignment of the *Petunia* homeodomain region of *WUSCHEL*-related homeobox genes. The helix-loop-helix-turn-helix homeodomain structure is indicated in the top, and the region of helix three not conserved in SOE is indicated below. Species names abbreviations are shown Table S1.
