# Supplementary material for: Contribution of WUSCHEL-related homeobox (WOX) genes to identify the phylogenetic relationships among Petunia species
Source: Genet Mol Biol. 2016 Oct 20;39(4):658–64. doi: 10.1590/1678-4685-GMB-2016-0073 (PMC5127159; doi:10.1590/1678-4685-GMB-2016-0073)
Supplement: Supplementary file 4 [file 1415-4757-gmb-1678-4685-GMB-2016-0073-Suppl02.pdf]

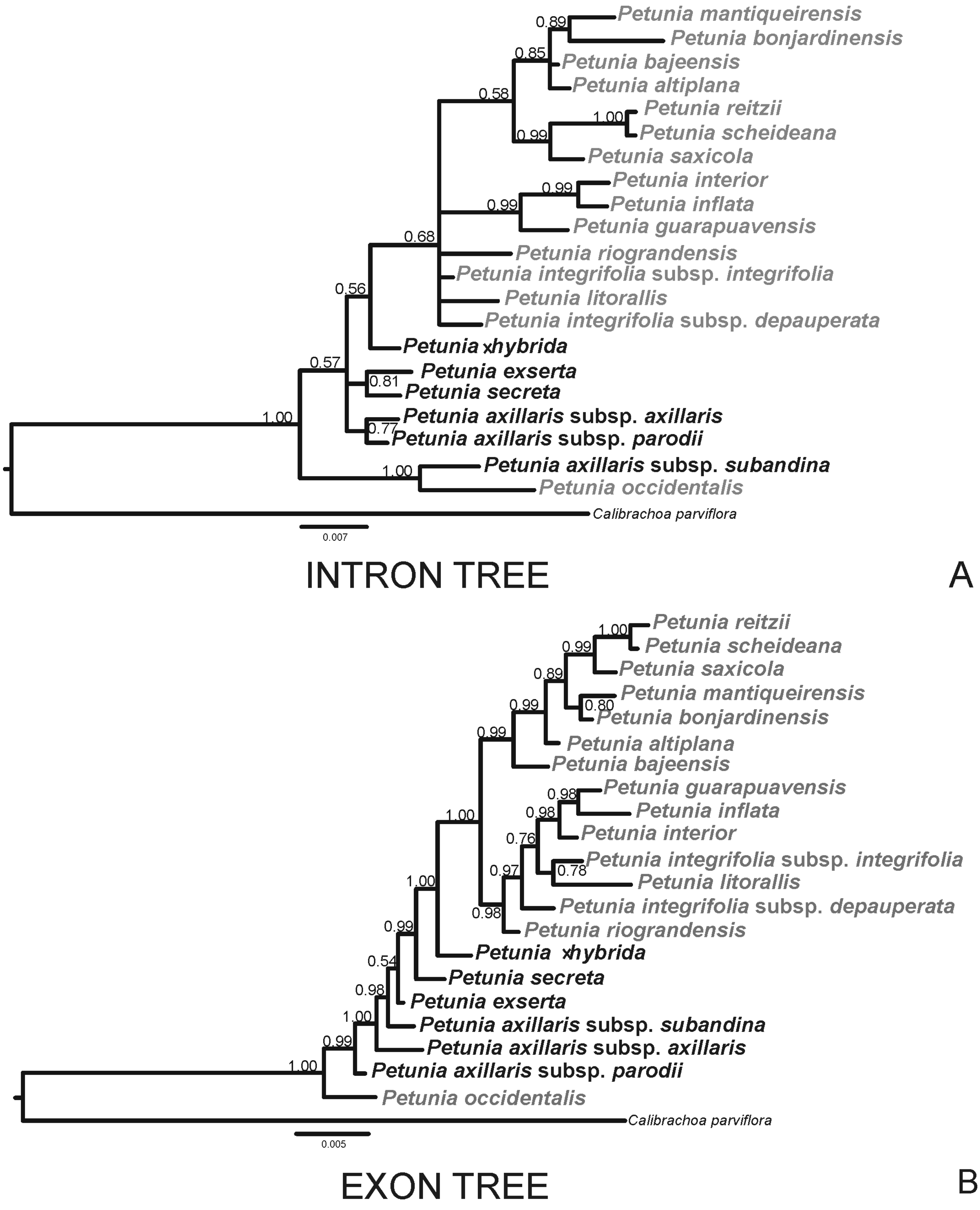

Figure S2 - Bayesian inference phylogenies considering the intron (A) or exon (B) sequences of *WUSCHEL*-related homeobox gene sequences in *Petunia* species. The short corolla tube species names are in gray and long corolla tube species are in black. The posterior probabilities are indicated.
